# Sources of Risk of AI Systems

**DOI:** 10.3390/ijerph19063641

**Published:** 2022-03-18

**Authors:** André Steimers, Moritz Schneider

**Affiliations:** Institute for Occupational Safety and Health of the German Social Accident Health Insurance (IFA), 53757 Sankt Augustin, Germany; moritz.schneider@dguv.de

**Keywords:** artificial intelligence, risk management, occupational safety, protective devices, assistance systems

## Abstract

Artificial intelligence can be used to realise new types of protective devices and assistance systems, so their importance for occupational safety and health is continuously increasing. However, established risk mitigation measures in software development are only partially suitable for applications in AI systems, which only create new sources of risk. Risk management for systems that for systems using AI must therefore be adapted to the new problems. This work objects to contribute hereto by identifying relevant sources of risk for AI systems. For this purpose, the differences between AI systems, especially those based on modern machine learning methods, and classical software were analysed, and the current research fields of trustworthy AI were evaluated. On this basis, a taxonomy could be created that provides an overview of various AI-specific sources of risk. These new sources of risk should be taken into account in the overall risk assessment of a system based on AI technologies, examined for their criticality and managed accordingly at an early stage to prevent a later system failure.

## 1. Introduction

Artificial intelligence (AI) methods are mainly used to solve highly complex tasks, such as processing natural language or classifying objects in images. AI methods do not only allow significantly higher levels of automation to be achieved, but they also open up completely new fields of application [1]. The importance of artificial intelligence is constantly increasing due to ongoing research successes and the introduction of new applications based on this technology. Driven by success in the fields of image recognition, natural language processing and self-driving vehicles, in the coming years, the fast-growing market of artificial intelligence (AI) will play an increasingly significant role in occupational safety [2,3].

Today, the term artificial intelligence is mainly used in the context of machine learning, such as decision trees or support vector machines, but also includes a variety of other applications, such as expert systems or knowledge graphs [4]. A significant subcategory of machine learning is deep learning, which deals with the development and application of deep neural networks. These neural networks are optimised and trained for specific tasks, and they can differ fundamentally in terms of their architecture and mode of operation [5]. An example would be the use of convolutional neural networks in the field of image processing [6].

AI systems are engineered systems that build, maintain, and use a knowledge model to conduct a predefined set of tasks for which no algorithmic process is provided to the system.

Thus, by using artificial intelligence, concepts such as learning, planning, perceiving, communicating and cooperating can be applied to technical systems. These capabilities enable entirely new smart systems and applications, which is why artificial intelligence is often seen as the key technology of the future [7].

Protective devices and control systems based on artificial intelligence have already enabled fully automated vehicles and robots to be created [8,9]. Furthermore, they enable accidents to be prevented by assistance systems capable of recognising hazardous situations [10,11].

However, for the benefit of human safety and health, safe and trustworthy artificial intelligence is required. This is because, despite the rapid, positive progression of this technology and the new prospects for occupational safety, the increasing application of this technology will also produce new risks [12]. Even today, we already face an increasing number of accidents in systems that utilise artificial intelligence [13], including various reports on fatal accidents due to AI-related failures in automated vehicles [14,15].

Established measures of risk reduction in the development of software are limited in their ability to mitigate these risks, and existing safety standards are hardly applicable to AI systems as they do not take into account their technical peculiarities [16]. For example, during verification and validation activities in the software life cycle, the influences of different input values in the system are investigated, but these can be relatively easily mapped by boundary value analyses. In the field of artificial intelligence, however, this is difficult due to the extensive and complex possible state space. These applications have to deal with the influence of many different biases [17], some of which are specific to AI systems and are therefore not considered in the required verification and validation activities of existing software safety standards.

For this reason, the development of safe AI systems requires a good understanding of the components of trustworthy artificial intelligence [18,19] as risk management for systems that utilise AI must be carefully adapted to the new problems associated with this technology.

Some research proposes assurance cases to support the quality assurance and certification of AI applications. These must provide assessable and structured arguments to achieve a certain quality standard [20,21,22].

However, these works lack a detailed list of concrete criteria. We propose to define these criteria based on the sources of risk for AI systems. These can then be analysed and evaluated within the risk assessment to derive appropriate risk mitigation measures; this proves to be necessary.

So, it is essential to identify these new risks and analyse the impact of AI characteristics on the risk management strategy, depending on the nature of the system under consideration and its application context. International standards for the AI field are mostly still under development and usually only address partial aspects such as the explainability [23] or controllability [24] of such systems or that they are not applicable to the field of safety-related systems [25]. Other legislative documents such as the Proposal for an Artificial Intelligence Act of the European Commission [26], by their very nature, only define generic requirements at a very high level from which relevant risk fields must first be derived.

Recent approaches for identifying and structuring specific sources of risk for AI systems have already identified some of these risks [12]. However, they do not yet consider important aspects such as security, nor do they offer a proposal for a structured process model for a complete risk assessment in the AI field or a complete taxonomy of risk sources, which would be necessary for the development of corresponding standards. Similarly, they give only a brief description of the sources of risk, which makes it difficult to gain a basic understanding of the difficulties associated with them.

Furthermore, care must be taken to ensure that all identified sources of risk are designed in such a way that they can be taken into account in the overall risk assessment of a system based on AI technologies, examined for their criticality and managed accordingly at an early stage to prevent a later failure of the system.

This paper addresses the question of how the risk of an AI application can be assessed in order not only to determine its level, but also to be able to effectively reduce it. In Section 2, a proposal for an AI risk management process is defined, which is based on established risk management standards, such as ISO 12100 [27] and ISO 14971 [28].

Section 3 then deals with an analysis and taxonomy of specific sources of AI risk. In particular, the relationship between AI technologies and their characteristics is analysed to raise awareness of the new challenges that this technology brings. Section 4 discusses the results presented and concludes by highlighting their significance.

## 2. Materials and Methods

In order to establish a strategy for promoting occupational safety and health that takes a particular technology into account, it is useful to look at the interaction of this technology with possible risks arising from it. It should be noted that there are different definitions of risk depending on the field of application. In general, risk is defined as the impact of uncertainty on objectives [29]. An impact can, therefore, be a deviation in a positive or negative direction, as well as in both directions. Accordingly, risks can also have positive effects, when following the definition of ISO 31000 [29].

In the context of safety-related systems, however, only the negative effects are usually considered at the system level, especially those that relate to the health and integrity of people. An example is given in the definition of risk according to the ISO IEC Guide 51 [30], which also serves as a basis for risk management standards, such as ISO 12100 [27] or ISO 14971 [28]. In these documents, risk is defined as the “combination of the probability of occurrence of harm and the severity of that harm”.

The discipline of occupational safety and health also uses the definition of ISO IEC Guide 51 [30], which is why this definition of risk is used in this paper. From a societal perspective, however, the more comprehensive definition of risk is also helpful and often useful.

Usually, risk is presented in terms of the cause of the risk, the potential events, their impact, and their probability [27,28]. Defined risk management processes are a common way of dealing with risks. These iterative risk management processes involve risk assessment and risk reduction. Risk assessment identifies sources of harm and evaluates the related risks for the intended use and the reasonably foreseeable misuse of the product or system. Risk reduction reduces risks until they become tolerable. Tolerable risk is a level of risk that is accepted in a given context based on the current state of the art.

In order to set up a risk management process for AI systems in the field of occupational safety and health, it is helpful to familiarise oneself with the standards mentioned above. ISO 12100 [27] is helpful here because it describes the risk management process for machinery. However, since AI systems are usually used for complex tasks in complex environments [31], and particularly deep-learning-based models are highly complex [32], the ISO 12100 [27] process needs to be modified somewhat to take these particularities into account.

The ISO 14971 [28] process is helpful in this regard, as active medical devices interact especially often with complex systems, in this case the human body.

Since this complexity results in certain uncertainties, and the complete testing of such systems with all possible interactions can be ruled out, field studies [33,34] are also used here in addition to other test measures in the verification and validation phase, but market monitoring [28] is also required in the risk assessment, in order to be able to carry out field safety corrective actions for distributed products if necessary. This measure is, therefore, a useful addition to the ISO 12100 [27] process, for example.

A resulting possible risk management process for AI systems that is general but still detailed is presented below:

1. Definition of risk acceptance criteria:
2. Risk assessment:
  2.1. Risk identification:
    2.1.1. Purpose;
    2.1.2. Identification of hazards (e.g., by FTA [35], FMEA [35], FMEDA [36]).
  2.2. Risk analysis:
    2.2.1. Extent of damage;
    2.2.2. Probability of occurrence;
      2.2.2.1. Hazard exposure;
      2.2.2.2. Occurrence of a hazard event;
      2.2.2.3. Possibility of avoiding or limiting the damage.
3. Risk evaluation:
4. Risk control:
  4.1. Analysis of risk governance options;
  4.2. Implementation of the risk control measures;
  4.3. Evaluation of the residual risk;
  4.4. Risk–benefit analysis;
  4.5. Analysis of the risks arising from risk governance measures;
  4.6. Assessment of the acceptability of the overall residual risk.
5. Market observation.

### 2.1. Definition of Risk Acceptance Criteria

The first step of a risk management process involves defining the risk acceptance criteria. In this step, the residual risk that is still acceptable or tolerable is defined. This residual risk is determined by the following factors, among others [37,38]:

- Extent of damage;
- Benefit;
- Voluntariness;
- Costs;
- Secondary effects;
- Delay of the damaging event.

The level of tolerable residual risk is, therefore, derived to a large extent from a tacit social consensus. An example of this is the use of nuclear energy in Germany. This technology has the potential to cause an enormous amount of damage, but at the same time offers a high level of benefit. The risk–benefit analysis was assessed either positively or negatively in political discourse, depending on which factor was weighted more heavily. A social consensus was found through corresponding parliamentary majorities. The occurrence of actual damaging events ultimately led to a short-term change in society’s position on this topic, which was promptly followed by an equivalent shift in thinking in the political field [39,40].

Today, there are still only a few recognisable social positions on artificial intelligence. Many applications are already being used and accepted subconsciously [41], while other applications are slowly gaining ground and are being more intensively discussed [42]. In areas where the application has a direct influence on people or could influence their health, the European market is still hesitant to accept AI technology [43]. The reasons for this are largely due to the lack of (comprehensive) regulatory provisions and the lack of corresponding technical standards to which they could refer. Where such systems are used, they are often misused [44,45]. This is based on a general lack of knowledge about the realistic possibilities of this technology. Public perception is usually determined either by promising utopian or dystopian scenarios. However, both of these notions are due to unrealistic perceptions of this technology, often stemming from the way it is portrayed in various media [46].

Therefore, for the widespread and socially accepted use of these technologies to be possible, it is necessary to create appropriate preconditions, which are closely linked to the definition of the risk acceptance criteria. The first steps in this direction were brought up by the development of normative foundations, i.e., in ISO IEC JTC 1 SC 42 “Artificial Intelligence” or CEN CLC JTC 21 “Artificial Intelligence”. Furthermore, the European Commission published a first proposal for a regulation on artificial intelligence [26].

Aside from the elaboration of regulative measures, it is equally helpful to inform the public about realistic application possibilities and the limits of this technology. Finally, the acceptance of the technology in a social context should be monitored.

### 2.2. Risk Assessment

The risk assessment consists of two elements: risk identification and risk analysis. First of all, the risk identification step defines the exact purpose of the application and its limits (compare to Determination of limits of machinery [27], Intended use in ISO 12100 [27], and identification of characteristics related to the safety of the medical device in ISO 14971 [28]). This step is of great importance in the field of artificial intelligence, but it is also difficult to implement, as this technology is mainly used in highly complex environments [31]. A peculiarity of these environments is that it is often not possible to completely define their boundaries, which in turn results in uncertainties. Therefore, one of the key questions regarding the use of artificial intelligence is the extent to which this technology can be used in a safety-related environment and how these uncertainties can be minimised. The answers to such questions can usually be found in corresponding regulations and standards. However, these do not yet exist and must be developed simultaneously to answering these questions.

In addition, all sources of risk associated with artificial intelligence must be identified. These include new sources of risk that can specifically occur with artificial intelligence methods, such as deep learning, but also classic sources of risk that contain new aspects in connection with the use of AI. These risk sources were investigated and will be presented in the results.

The subsequent risk analysis finally examines the probability of occurrence and the actual hazard exposure for each individual identified risk (compare to Identification of hazards and hazardous situations/Hazard identification and Risk estimation/Estimation of the risk(s) for each hazardous situation in ISO 12100 [27] and ISO 14971 [28]). Since there often remains little experience in the development and the use of applications based on this technology—which, in turn, means that the handling of the associated risks is usually of an unknown quantity—small- and medium-sized companies that have not already addressed the area of Trusted AI need extensive assistance in the long term.

When conducting a risk assessment of a workplace, its risk is essentially determined by the following three factors [27]:

- Hazard exposure;
- Occurrence of a hazardous event;
- Possibility of avoiding or limiting the harm.

Section 3 (the Results section) describes various AI-specific risk factors for consideration in a risk assessment. These can be analysed and assessed in the context of the specific AI system. If an unacceptable risk is identified, appropriate risk reduction measures tailored to the individual sources of risk described can then be defined and applied.

### 2.3. Risk Evaluation

The risk evaluation is based on the results of the risk assessment, which evaluates the existing or potential risk with regard to the extent of damage and the probability of occurrence on the one hand and its impact on the application on the other. This step is often carried out together with a third party in order to have a neutral and independent opinion on this critical step of the product life cycle [27,28].

### 2.4. Risk Control

After the first iteration of the previous steps, the preliminary result of the risk assessment is determined. If this shows that the tolerable risk is exceeded, risk control measures must be applied. After analysing the options for risk control, these must finally be implemented, and the residual risk must be reassessed (compare to Risk reduction in ISO 12100 [27] and Risk control in ISO 14971 [28]).

Usually, risk control measures are hierarchically prioritised. For example, ISO IEC Guide 51 [30] establishes a three-level classification:

1. Inherently safe design;
2. Safeguards and protective devices;
3. Information for end users.

In general, an inherently safe design should always be attempted; in cases where this is not possible, safeguards and protective devices can be used. If all of these measures are not possible, information for the end users is mandatory.

The problem is that the transfer of a concept idea or an existing product to a safety-related application is a difficult undertaking that requires a lot of experience. Not only are the existing regulations and standards a hurdle, but the concrete implementation of measures also poses a significant challenge.

Technical measures are based on the four pillars of inherently safe design, safety reserves, safe failure and safety-related protective measures. In the field of artificial intelligence, however, these have some special features that need to be considered [27].

#### 2.4.1. Inherently Safe Design

In machine learning, the quality of the result depends to a large extent on the quality of the training data. If the training data do not cover the full variance of the test data or contains errors, the model will produce an equally erroneous algorithm [47]. If a very complex model is used, it is very difficult to understand the decision-making process of the algorithm, and thus identify faulty parts of the software. Consequently, it is advantageous to choose models with a low level of complexity that can be interpreted by humans and can, therefore, be checked and maintained. In this way, features that do not contribute to a causal relationship with the result, and would, therefore, lead to erroneous results, can be removed manually. A disadvantage of interpretable models, however, is that their simplicity is often accompanied by a lower quality in terms of the probability of a correct result [48,49,50].

#### 2.4.2. Safety Margins

When we look at a mechanical system, for example, there is a point at which a load leads to the failure of the system. As this point can usually only be determined within a certain tolerance range, these systems are operated far below these limits by introducing a certain safety margin or safety factor.

Such uncertainties can also be identified in machine learning. For example, there is uncertainty about whether the learning dataset completely covers the distribution of the test data or uncertainty regarding the instantiation of the test data. Insofar as this uncertainty is captured, a safety margin or safety limit range can also be defined for an algorithm that sufficiently delimits the areas of a reliable decision from those in which an uncertainty exists. Therefore, models that can algorithmically calculate a measure for the uncertainty of their prediction are to be preferred [51,52].

For classification problems, for example, the distance from the decision boundary can be used, whereby a large distance means an increase in the reliability of a prediction [53]. At the same time, however, it must be noted that this only applies to areas in which a high number of available training data exists, and thus have a high probability density. The reason for this is that, in areas with a low probability density, there is usually little or even no training data available. This leads to the fact that, in these areas, the decision boundary is determined by inductive errors, and thus a high epistemic uncertainty, which means that the distance from this boundary has no significance with regard to the reliability of the prediction [54].

#### 2.4.3. Safe Failure

One of the most important strategies in safety engineering is the principle of safe failure. Again, it is important to have a measure of the uncertainty of the prediction. If this is relatively high, the system could request further verification by a human. In the case of a collaborative robot, however, this would also mean that the robot arm would first have to assume a safe state [52].

#### 2.4.4. Safety-Related Protective Measures

Safety-related protective measures can be implemented in a variety of ways and cover a broad spectrum, from external protective devices to quality-assuring processes for error minimisation. The development process for software in the safety-related environment is governed by a wide range of regulations and standards. The IEC 61508 series of standards—“Functional safety of safety-related electrical/electronic/programmable electronic systems”, Part 3 “Software requirements” [36]—is a good example of this. This standard also contains many methods that can be applied to avoid and reduce systematic errors during software development. This development process is embedded in the Functional Safety Management (FSM) plan, which, among other things, describes the entire lifecycle of the system in Part 1. In addition, there are some software-related requirements in Part 2 of this series of standards that must also be considered. However, to date there are no regulations that clarify the relationship between functional safety and artificial intelligence or describe special measures for AI systems in a safety-related environment. At the international level, since 2020, initial activities have been underway to describe requirements for the use of artificial intelligence in the context of functionally safe systems [55].

### 2.5. Market Observation

New technologies, products and applications can bring with them new risks that, in the worst case, can be overlooked or underestimated. In order to identify such risks, consider them in the future or be able to remove a product from the market in time to improve it, it is necessary to observe the market and to analyse negative incidents in connection with the respective product. For this purpose, not only is it necessary to collect and review reports from the public media, but the specialist literature must be also consulted. Furthermore, an analysis of corresponding accident data would be useful for prevention purposes [28] (ISO 13485).

## 3. Results

The overall risk management process describes a procedure that identifies risks, assesses them, and defines measures to control them. The core of this process is, of course, the risk assessment, as it is here that the prevailing risks for the system at hand are identified and analysed. To be able to assess the hazards emanating from a technical system, a precise analysis of the sources of risk associated with this system is required. For example, the ISO 12100 standard, “Safety of machinery—General principles for design—Risk assessment and risk reduction” [27], specifies the principles of risk assessment and risk reduction for the machinery sector. This standard contains general sources of risks to be assessed for the machinery sector, whereas the standard ISO 14971, “Medical devices—Application of risk management to medical devices” [28], describes principles of risk management in the sector of medical devices.

However, the use of new technologies, such as the various methods used in the field of artificial intelligence, brings new specific sources of risk or gives rise to new aspects of existing sources of risk that need to be assessed. It is therefore of great importance to identify these sources of risk so that they can be assessed as part of the risk assessment of an AI system.

To identify these new AI-specific sources of risk, it is necessary to evaluate various sources. First, current research trends are relevant. In the field of artificial intelligence, the success of probabilistic and statistical approaches to machine learning in recent years has been undeniable [56,57], and interest in this area continues to be unabated [58]. For this reason, these methods need to be analysed in detail to obtain a list of risk sources that have an impact on the safety of AI systems. For example, the ongoing scientific discussion on the topic of XAI (explainable artificial intelligence) shows that this is one of the core problems of deep learning [59,60,61]. This problem is a direct result of model complexity, which in turn results from the application of artificial intelligence to complex tasks in complex environments [62,63]. In this context, however, the question is not only to what extent a task can be automated, but also to what extent it should be automated [64,65]. For example, Article 22 of the General Data Protection Regulation of the European Union [66] states that “The data subject shall have the right not to be subject to a decision based solely on automated processing…”. Overall, it can be said that privacy issues are particularly important when using machine learning methods, as these are based on the collection and processing of large data sets [67,68,69]. Security aspects can also have an impact on the safety of the system, making it important to assess the integrity of the safety behaviour against intentional inputs such as attacks. These include, for example, known inputs that destroy the integrity of software execution (e.g., buffer overflow) but also specific inputs that cause AI models to compute poor results without causing software-level malfunctions [70,71,72]. Another problem comes from automated decision-making systems, as they run the risk of being subject to a bias that leads to discriminatory results. For this reason, fairness is a major issue [73,74,75,76,77]. One problem with any new technology is the lack of experience in using it; a system that is proven in use can usually be trusted more, as it is assumed that any weaknesses have been discovered and fixed and that it has proven itself to be functional. This lack of experience can also be a problem when using new AI procedures. On the other hand, it represents an opportunity to make the general complexity of these systems controllable (cf. proven in use acc. IEC 61508 [36]).

After an analysis of the research literature, from which various sources of risk could be derived, studies were first compared with existing research on measures for the quality assurance of AI systems. The work of Houben et al. [78] should be mentioned, which provides a comprehensive study of measures for realising the safety integrity of AI systems. Based on the research of such quality-assurance measures, the fundamental problem areas addressed by these measures, and whether they correspond to the previous collection of identified risk sources, were investigated. Subsequently, existing regulations and research on the certification of AI applications were examined. Here, the work of Mock et al. [79], for example, provides a broad overview of the requirements from the draft of an AI regulation of the European Union [26], some research by the ISO/IEC, and the High-Level Expert Group of the EU Commission [19]. Furthermore, this work provides a direct comparison between these documents, which makes it possible to check whether the risks identified so far address these requirements. This comparison was complemented by the current work of Working Group 3 of the standardisation body ISO/IEC JTC 1 SC 42. As a result of these steps, the technical risk sources identified so far were complete according to this work. It should be noted, however, that these differed in part in their terminology as well as in their content. For example, “safety” was often directly addressed in the work discussed. However, to our understanding, this property or basic requirement is a result of a reliable and robust system, which in turn is a result of requirements from various other sub-items. Furthermore, this work does not consider legal risks but focuses on technical requirements and measures for the realisation of a safe AI system.

Finally, to complete the evaluation of the identified sources of risk, the risks were applied to various fatal accidents in recent years [14,15]. The premise was that a risk assessment based on an investigation of the aforementioned sources of risk would have had to address the technical deficiencies revealed by the follow-up investigation of these accidents. The previously identified sources of risk proved themselves in this analysis; nevertheless, it turned out that one critical source of risk was missing. For example, some accidents were based on weaknesses in the system hardware [80], which should be included as a source of risk in AI systems, especially since AI-specific peculiarities also exist here.

In order to be able to classify the identified sources of risk in a taxonomy, the components of a trustworthy AI, according to the High-Level Expert Group on Artificial Intelligence (AI HLEG) of the European Commission, should be used as a guideline. According to the “Ethics guidelines for trustworthy AI” of the AI HILEG, trustworthy AI comprises three components, entailing that the actors and processes involved in AI systems (including their development, deployment and use) should be:

- Lawful—complying with all applicable laws and regulations;
- Ethical—ensuring adherence to ethical principles and values;
- Robust—both from a technical and social perspective.

As mentioned, this work deals with purely technical sources of risk or those sources of risk that entail a technical implementation and not with legal issues.

**Figure 1:**
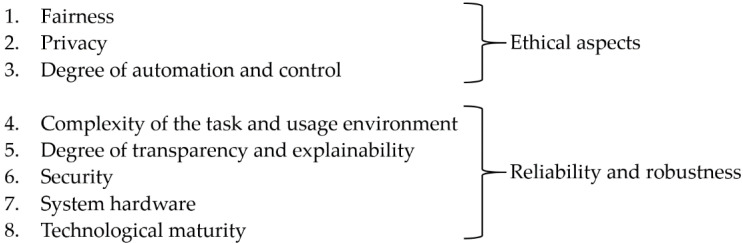
Sources of risk in AI systems that impact the trustworthiness of the system.

On this basis, the taxonomy of different sources of risk that can influence the trustworthiness of an AI system presented in Figure 1 was drawn up.

These can be roughly divided into two different blocks. The first block deals with ethical aspects. These include fairness, privacy and the degree of automation and control.

The second block deals with various aspects that can influence the reliability and robustness of the AI system, and thus have a direct influence on the safety of the system. Generally, robustness relates to the ability of a system to maintain its level of performance under any circumstances of its usage [4]. Robustness differs from reliability in that a reliable system only needs to maintain its level of performance under the specified conditions for a specific period of time [4]. Robustness, on the other hand, also includes stability against bias or errors and, therefore, represents an extension of the concept of reliability.

In the case of AI, robustness properties demonstrate the ability of the system to maintain the same level of performance when using new data as it achieves when using the data with which it was trained or data for typical operations. Robustness is a new challenge in the context of AI systems, as these systems are used for very complex tasks in complex usage environments, which involve a certain degree of uncertainty. Neural network architectures represent a particularly difficult challenge, as they are both hard to explain and sometimes have unexpected behaviour due to their nonlinear nature. Furthermore, some machine learning methods offer new attack vectors that can reduce the security of the system against external attacks. It is also important to consider the multiple influences of hardware failures, as well as the specific aspects related to them, which can also have a negative effect. Finally, the technological maturity of the AI method used is another important aspect to consider.

Details of the above properties and risk factors, along with their related aspects and challenges, are discussed below.

### 3.1. Fairness

The general principle of equal treatment requires that an AI system upholds the principle of fairness, both ethically and legally. This means that the same facts are treated equally for each person unless there is an objective justification for unequal treatment.

AI systems used for automated decision-making pose a particular risk for the unfair treatment of specific persons or groups of persons.

To ensure a fair AI system, it must first be investigated whether or to what extent the specific system could make unfair decisions. This depends on various factors, such as the intended use of the system, as well as the information available for decision-making. If the decisions made by the system cannot have an effect on either natural or legal persons or if the system has no information to distinguish between individuals or groups, it can be assumed that the system does not pose a high risk of discrimination.

The next step is to identify those individuals or groups who can potentially be disadvantaged by the AI system. These can be social minorities or socially disadvantaged groups, but also companies or legal entities in general, as is the case with pricing in digital marketplaces, for example. The General Equal Treatment Act describes various groups of natural persons that are subject to the risk of discrimination:

- Persons of a certain nationality;
- Persons of a certain ethnic origin;
- Persons of a particular gender;
- Persons belonging to a particular religion or belief;
- Persons with a physical or mental disability;
- Persons belonging to a certain age group;
- Persons with a particular sexual identity.

The relevance of these hazards must be assessed in relation to the AI system under investigation. In addition, other groups of people must be considered and added if they could be discriminated against due to the specific context of use or the requirements of the AI application. Likewise, depending on the use case, it must be assessed whether discrimination against legal persons could occur.

If one or more potential hazards to specific individuals or groups are identified, measures must be taken to reduce them to an acceptable level. To do this, it is helpful to look at the causes of possible discrimination.

Unfair outcomes can have several causes, such as bias in objective functions, imbalanced data sets and human biases in training data and in providing feedback to systems. Unfairness might also be caused by a bias issue in the system concept, the problem formulation or choices about when and where to deploy AI systems.

Generally, fairness can be considered as the absence of discrimination in the decisions of an algorithm or in a dataset. For systems whose models were developed on the basis of machine learning methods, the quality of the database is a particularly decisive factor. Here, particular attention must be paid to both the balance and the diversity of the data. Rare cases must not be underrepresented here. Measures that can be used to ensure that datasets are sufficiently balanced and diverse include the massaging, reweighing or sampling of the data.

To prove that an AI system acts fairly, it is necessary to use objective criteria. Fairness metrics are particularly useful here. Such metrics exist for both groups and individuals.

The metrics of group fairness indicate whether all groups of people are treated fairly. When a group is prone to be discriminated against by society, it is referred to as the protected group, whereas a group that is not vulnerable to discrimination is referred to as an unprotected group.

- **Calibration**
  This states that, for any prediction, samples originating from the protected group must have the same odds to be predicted positively as samples originating from the unprotected group [81].
- **Statistical parity**
  Statistical parity, also referred to as demographic parity, means that the predictions should be independent of whether a sample belongs to the protected group or not, i.e., the demographics of the set of individuals receiving any classification are the same as the demographics of the underlying population as a whole [82,83,84,85].
- **Equalised odds**
  This states that the true positive rates and false positive rates of the outcomes should be the same for the protected group as for the unprotected group. Intuitively, the number of samples classified in the positive class should be the same for the protected group as for the unprotected group [86,87].
- **Equality of opportunity**
  This is a relaxed version of the equalised odds condition, where it is only required for both the protected and the unprotected group to have an equal true positive rate [88,89].
- **Conditional statistical parity**
  This states that over a limited set of legitimate factors, the predictions for a sample should be the same for every group. This can be seen as a variant of statistical parity, in which factors that might explain some justified differences in the predictions for two groups are taken into account [85].
- **Predictive equality**
  This states that decisions made on the protected and unprotected groups should have the same false positive rate. This metric also relates to the equalised odds and equality of opportunity metrics [85].
  The metrics of individual fairness focus on individuals and the total set of characteristics that define them, instead of focusing on one characteristic that dictates which group they belong to.
- **Fairness through unawareness**
  This states that an algorithm is fair if any protected attributes are not explicitly used in the decision-making process. This approach to fairness is also referred to as suppression of the protected attributes [88].
- **Fairness through awareness**
  This states that an algorithm is fair if similar individuals are treated similarly. By similarity, it is meant that many of their defining characteristics are the same. For every classification task, a similarity measure must be defined. The objective of this measure is to define what exactly is meant by similarity between individuals in the specific context of that task [82].
- **Counterfactual fairness**
  This states that a decision is fair towards an individual if it coincides with the decision that would have been taken in a counterfactual world. A counterfactual world refers to a situation in which the sensitive attribute is flipped (e.g., flipping the gender). This results in a situation where the individual has the exact same characteristics, apart from the fact that they now belong to the unprotected group instead of the protected one [88].

### 3.2. Privacy

Privacy is related to the ability of individuals to control or influence what information related to them may be collected and stored and by whom that information may be disclosed. Due to their characteristics, it is possible for AI applications to interfere with a variety of legal positions. Often, these are encroachments on privacy or the right to informational self-determination.

Many AI methods process a variety of different data. Machine learning methods and deep learning methods are especially dependent on large amounts of data, as they need sufficient data to train their models. Ultimately, their accuracy often correlates with the amount of data used. The misuse or disclosure of some data, particularly personal and sensitive data (e.g., health records), could have harmful effects on data subjects. For example, AI applications often process sensitive information, such as personal or private data, including voice recordings, images or videos. Therefore, it must be ensured that the respective local data protection regulations, such as the General Data Protection Regulation (GDPR) [66] in Europe, are observed and complied with.

However, not only can AI applications endanger the privacy of a natural person, but also that of legal persons, for example by releasing trade secrets or license-related data.

Since AI systems often combine data that were previously not linked, it is often possible for them to even capture complex relationships through the creation of extensive models, and thus directly identify persons without even directly specifying the corresponding attributes. However, in addition to the stored or processed data, the ML model implemented in the AI application can also be spied out, which in turn would allow an attacker to extract personal (training) data from a model.

Therefore, privacy protection has become a major concern in Big Data analysis and AI. Considerations regarding whether or not an AI system can infer sensitive personal data should be taken into account. For AI systems, protecting privacy includes protecting the training data used for developing the model, ensuring that the AI system cannot be used to give unwarranted access to its data, and protecting access to models that have been personalised to an individual or models that can be used to infer information on characteristics of similar individuals.

The risk assessment must determine which specific threats to personal data are posed by the AI system. In particular, the type and significance of the data retrieved or stored during the product lifecycle must be investigated and potential gaps in protection must be identified. It should be noted that this applies not only to data used for development, but also to data used during operation.

The handling of personal data is regulated, for example, by the European General Data Protection Regulation. It should be noted that the legal requirements are not only violated by unauthorised access by third parties, but by the mere existence of unauthorised access, as well as inappropriately long storage periods, and the impossibility to obtain information about the stored data.

Article 5, paragraph 1 of the GDPR [66] describes several principles relating to processing personal data in this regard:

- **Lawfulness, fairness, and transparency**
  Personal data shall be processed lawfully, fairly and in a transparent manner in relation to the data subject.
- **Purpose limitation**
  Personal data shall be collected for specified, explicit and legitimate purposes and not further processed in a manner that is incompatible with those purposes.
- **Data minimisation**
  Personal data shall be adequate, relevant and limited to what is necessary in relation to the purposes for which they are processed.
- **Accuracy**
  Personal data shall be accurate and, where necessary, kept up to date; every reasonable step must be taken to ensure that personal data that are inaccurate, having regard to the purposes for which they are processed, are erased or rectified without delay.
- **Storage limitation**
  Personal data shall be kept in a form which permits identification of data subjects for no longer than is necessary for the purposes for which the personal data are processed.
- **Integrity and confidentiality**
  Personal data shall be processed in a manner that ensures appropriate security of the personal data, including protection against unauthorised or unlawful processing and against accidental loss, destruction, or damage, using appropriate technical or organisational measures.

If risk control requirements arise, various measures exist to preserve the privacy of personal data. For example, data can be anonymised or pseudonymised. A perturbation or aggregation of data in modelling can also be an effective means of preserving privacy. In general, care should be taken to ensure that only data for a specific purpose are used to the extent necessary.

Another way to prevent unwanted access to data is federated learning, in which several models are trained locally on different computer nodes, so that the respective training data do not have to leave their local position. The separately generated models are then combined into a global model.

### 3.3. Degree of Automation and Control

The degree of automation and control describes the extent to which an AI system functions independently of human supervision and control.

It thus determines not only how much information about the tactile behaviour of the system is available to the operator, but also defines the control and intervention options of the human. On the one hand, an assessment is made with regard to how high the degree of automation must be for the respective application, but on the other hand, an assessment is also made with regard to whether the human is adequately supported by the AI application and is given appropriate room for manoeuvring in interactions with the AI application. Systems with a high degree of automation may exhibit unexpected behaviour that can be difficult to detect and control. Highly automated systems can, therefore, pose risks in terms of their reliability and safety.

In this context, several aspects are relevant, such as the responsiveness of the AI system, but also the presence or absence of a critic. In this context, a critic serves to validate or approve automated decisions of the system.

Such a critic can be realised through technical control functions, for example by adding second safety instruments for critical controls that can be understood as an assignment of safety functions to redundant components in the terms of the functional safety standards like IEC 61508-1 [36]. Another way of adding a critic is to use a human whose task is to intervene in critical situations or to acknowledge system decisions. However, even if humans are in the loop and control the actions of a system, this will not automatically reduce such risks and may introduce additional risks due to human variables such as reaction times and understanding of the situation.

Furthermore, the adaptability of the AI system must be considered. Here, the question of whether or to what extent the system can change itself must be considered. Systems that use continuous learning in particular change their behaviour over time. These systems have the advantage of acquiring new functions or adapting to changing environmental conditions via feedback loops or an evaluation function. The disadvantage of such systems, however, is that they can deviate from the initial specification over time and are difficult to validate.

In general, there is a tension between the autonomy of humans and the degree of automation of the AI system. As a rule, a high degree of automation restricts the possibilities of control and influence, and thus, ultimately, the autonomy of humans. It should, therefore, be ensured that human action always takes precedence when using an AI system, i.e., that the human being is always at the centre of the application.

This results in the task of creating an appropriate and responsible distribution of roles between humans and the AI system during the development of such a system. The best way to achieve this is by involving future users as well as domain experts in the development process. The degree of automation must be appropriate to the application context and provide the necessary control options for users. This will ultimately result in a human-centred AI.

In particular, the area of human–machine interaction is the focus for the use of a high degree of automation. AI systems are already being used today in many safety-related applications such as self-driving vehicles [90], aviation [91,92] or the operation of nuclear power plants [93]. In these areas, it is particularly important to ensure that system controls are understandable to people and behave in operation as they would during the design phase.

However, this raises the question of how to manage the uncertainties associated with human–machine interaction with AI-based systems [94]. If human–machine interaction leads to errors or injuries, the question of responsibility arises, as this may be due to incorrect input from the operator but also incorrect or contradictory sensor data. Moreover, in a highly automated system, there is only a limited possibility of human control over the automated system [95,96]. On the other hand, it is also questionable under what conditions an AI system can take control whilst avoiding injuries or errors.

Degrees of automation can be divided into seven different levels, starting from no automation at level 0 to an autonomous system at level 7, which represents the highest level of automation. The SAE standard J3016 [97] defines only six levels for the automotive sector, whereas the standard ISO/IEC 22989 [4] introduces the mentioned seven levels and provides a general description.

It should be noted, however, that today’s systems are still all in the area of heteronomous systems, and thus, in practical implementation only a maximum automation level of 6 (full automation) is currently feasible. Table 1 provides an overview and description of the different degrees of automation. The figure also shows how the degree of control by humans decreases as the degree of automation increases.

There is some confusion amongst the public, including developers, about the concept of autonomy in the context of AI systems. In general, it must be noted that it is not yet possible to produce artificial autonomous systems by technical means. AI systems as we find them today, can still all be classified as heteronomous systems. Heteronomous systems are distinguished from autonomous systems by being governed by external rules or the fact that they can be controlled by an external agent. In essence, this means that they are operated using rules that are defined or validated by humans. In contrast, an autonomous system is characterised by the fact that it is a system governed by its own rules and not subject to external control or oversight.

A common misconception today is that, in machine learning, the system creates its own rule set, and thus meets the definition for an autonomous system [47,98]. However, it is important to note that these rules are by no means created entirely by the system itself, but rather by the specification of a human-defined algorithm and a training data set determined by the human. Furthermore, these rules are developed to solve a specific task that is also specified by the human. Therefore, in this process, the human not only has complete supervision, but also far-reaching control possibilities.

The concept of autonomy is much broader. In Kant’s moral philosophy, for example, the concept of autonomy is defined as the capacity of an agent to act in accordance with objective morality and not under the influence of desires [99]. The concept of autonomy is, therefore, very closely linked to the concept of ethics and, ultimately, to the concept of free will. It is obvious that, to date, there are no AI systems that could be said to have free will, as all AI systems are still completely deterministic systems.

In their work entitled *Moral Machines*, Wallach and Allen [100] specifically address the concept of potentially autonomous machines and view them in the direct context of their ethical sensitivity. They distinguish between operational morality, functional morality and full moral agency. A system has operational morality when the moral significance of its actions are entirely determined by the designers of the system, whereas a system has functional morality when it is able to make moral judgements when choosing an action, without direct human instructions. A fully autonomous system would be a moral agent that has the ability to monitor and regulate its behaviour based on the harm its actions may cause or the duties it may neglect. Thus, a moral actor can not only act morally, but it can also act according to its own moral standards, which means that it would be able to form its own ethical principles or rules. Looking at the capabilities of today’s AI systems, only the level of operational morality can be implemented, so the requirement for an autonomous system is not met.

Wallach and Allan [100] also look at various important abilities that contribute to human decision-making, such as emotions, sociability, semantic understanding and consciousness. As these abilities contribute to human decision-making, they are basic prerequisites for moral systems. If we take only the point of perception here and compare the individual properties associated with human perception with AI systems, it can also be seen that these do not yet fulfil the necessary requirements for autonomous systems. This is summarised again in Table 2.

### 3.4. Complexity of the Intended Task and Usage Environment

AI is sensibly used for tasks for which there are no classic technologies as alternatives. Such tasks are usually characterised by a high degree of complexity. This complexity can arise from the task itself, as is the case, for example, with the classification of people, or from the complexity of the environment in which it is used, as is the case, for example, in the field of self-driving vehicles. Often, both apply evenly, which makes the task even more difficult.

This complexity gives rise to a certain level of uncertainty in the system’s behaviour, which is often perceived as non-deterministic, but whose cause lies in the fact that a complex task and/or environment can only be analysed and described completely by a human with great difficulty. This is mainly due to the large state space of such an environment, which can also be subject to constant change, which, in turn, continuously enlarges the state space, whereby it can be assumed that even a model that generalises the state space very well will not react appropriately to every possible state of the environment.

At this point, however, it should be pointed out once again that AI systems fundamentally work deterministically, even if it may appear otherwise for the reasons mentioned above. The only exceptions are systems that are based on continuous learning and whose models can adapt further during operation.

However, this uncertainty also means that, in the area of safety-related systems, it must be carefully examined whether it is absolutely necessary to use machine learning methods, and in particular deep learning, in the creation of the safety-related system, or whether this could also be carried out using alternative (AI) technologies.

The complexity of the intended task and usage environment of an AI system determines the full range of possible situations that an AI system, when used as intended, must handle. Since it cannot be assumed that it is possible to carry out an accurate and complete analysis of the environment and task to produce the system specification, it will inevitably become relatively vague and incomplete. This may result in the actual operating context deviating from the specified limits during operation.

As a general rule, more complex environments can quickly lead to situations that had not been considered in the design phase of the AI system. Therefore, complex environments can introduce risks with respect to the reliability and safety of an AI system.

For this reason, an AI system must have the ability to still provide reliable results even under small changes in input parameters. Although it is often not possible to predict all possible states of the environment that an AI system may encounter during its intended use, efforts should be made during the specification phase to gain an understanding of the intended use environment that is as complete as possible. In doing so, knowledge about the input data underlying the decision-making processes and the sources used to obtain it, such as the sensor technology used, should also be obtained and considered. Important aspects here are the questions of whether the system is fed with deterministic or stochastic, episodic or sequential, static or dynamic, and discrete or continuous data.

In the implementation phase, the system is built according to the requirements of the specification. For this purpose, the specification is usually analysed and interpreted by a development team to create a strategy for the technical realization of the system. This strategy and the associated design should again be kept as simple as possible to reduce the complexity of the system and increase its transparency. The implementation strategy is of great importance here, as it can have an immense impact on the design. For example, a requirement can often be paraphrased in such a way that it is still fulfilled semantically, but its technical implementation can be significantly simplified. As an example, consider the simple requirement for a collaborative robot arm that should not reach for a human. This function can already be implemented relatively reliably using deep learning methods, but it is a very complex task for a technical system because the possible state space for the object “human” is very large. If, however, this requirement is formulated in such a way that the robot arm may only grip a certain selection of workpieces, the original goal of the basic requirement is likewise fulfilled but now represents a fairly easy output for the technical system to handle since the object “workpiece” can be completely specified, and thus spans a very small state space.

Another special feature of AI systems based on machine learning methods is the basic implementation process. In classical software development, the specification is interpreted by the development team and implemented accordingly. However, in machine learning systems, which are trained with the help of an algorithm based on data, the mental concept of the specification must be described implicitly by the database. Therefore, the composition of the database and the general data quality are of immense importance. Furthermore, the training algorithm does not always find the best possible solution, which is why it is usually necessary to invest a large amount of work in the optimisation of the resulting model and is standard practice to complete many training runs with different parameterisation, in order to receive a model that is as effective as possible.

Reusing existing components, modules, trained models or complete systems in a new application context can lead to problems due to the different requirements between the specified context and the new context. For example, the use of a system designed to identify people in photos on social networks cannot be easily used to identify people in the context of an assistance system in a work environment. This is partly due to the different state spaces of the applications, so the system may not be able to recognise workers in their personal protective equipment because there are no data to train the model for this but also due to the higher precision required for the latter application. This shows that even a transfer to new environments, for example other industries, is not easily possible.

A few selected examples for model-specific problems regarding the use of trained agents or reinforcement learning are, in no specific order, reward hacking or the safe exploration problem.

The term reward hacking refers to a phenomenon where AI finds a way to gain its reward function, and thus finds a more optimal solution to the proposed problem. This solution, while being more optimal in the mathematical sense, can be dangerous if it violates assumptions and constraints that are present in the intended real-world scenario. For example, an AI system detecting persons based on a camera field might decide that it can achieve very high rewards if it constantly detects persons, and thus will follow them around with its sensors, potentially missing critical events in other affected areas. This can be countered by employing adversarial reward functions, for example, an independent system that can verify the reward claims made by the initial AI and can, most importantly, learn and adapt. Another option is to pre-train a decoupled reward function that is based solely on the desired outcome and has no direct feedback relation to the initial AI during training.

The safe exploration problem is of particular concern when an agent has the capability to explore and/or manipulate its environment. It is important to note that this does not only pose a problem when talking about service robots, UAVs or other physical entities, but also applies to software agents using reinforcement learning to explore their operating space. In these contexts, exploration is typically rewarded, as this provides the system with new opportunities to learn. While it is obvious that a self-learning AGV needs to follow proper safety protocols when exploring, a system that controls process parameters and employs a random exploration function while not being properly disconnected from the actual process (e.g., via simulation) can pose equal or greater safety risks.

Following proper safety precautions, the first step in ensuring safe operation is typically the application of supervision functions that take over the system in the event that a safety risk is detected, thereby ensuring that no harm can be carried out by the AI. Other options include encoding safe operations as part of the reward function of the system, for example by instructing the model not only to minimise the distance travelled for an AGV, but also to maximise distance to persons. That way, safety precautions become an intrinsic concern of the model by means of the actual reward function.

To cope with the complexity of the task and environment, data quality plays an important role in systems based on machine learning methods. The data must not only be complete, but also diverse, and thus representative enough that a suitable model can be generalised from them. In addition to these two very basic requirements for data quality, there are several other characteristics that must be maintained in order to ensure high data quality:

- **Accuracy**
  Accuracy is the degree to which data have attributes that correctly reflect the true value of the intended attributes of a concept or event in a particular context of use.
- **Precision**
  Precision is the extent to which data have attributes that are accurate or allow discrimination in a particular context of use. Precision refers to the closeness of repeated measurements to each other for the same phenomenon, i.e., the extent to which random errors contribute to the measured values.
- **Completeness**
  Completeness refers to the extent to which a data set contains all of the data it needs to contain.
- **Representativeness**
  Representativeness refers to the extent to which a data set representing a sample of a larger population has statistical properties that match the properties of the population as defined by the representative sample.
- **Consistency**
  Consistency refers to the extent to which multiple copies of the same data set contain the same data points with the same values.
- **Relevance**
  Relevance refers to the extent to which a dataset (assuming it is accurate, complete, consistent, timely, etc.) is appropriate for the task at hand.
- **Data scalability**
  Data scalability indicates the extent to which data quality is maintained as the volume and velocity of data increases.
- **Context coverage**
  Context coverage is the degree to which data can be used both in the specified contexts of an ML algorithm and in contexts beyond those originally explicitly specified.
- **Portability**
  Portability is the degree to which data have attributes that allow them to be installed, replaced or moved from one system to another, while maintaining their existing quality in each context of use.
- **Timeliness**
  Timeliness indicates the extent to which data from a source arrive quickly enough to be relevant. Timeliness refers to the latency between the time that a phenomenon occurs and the time the data recorded for that phenomenon are available for use; this dimension of data quality is particularly important when the dataset is a continuous stream of data.
- **Currentness**
  Currentness is the extent to which data have attributes that are the correct age in a particular context of use.
- **Identifiability**
  Identifiability is the extent to which data can be identified as belonging to a particular person, entity or small group of persons or entities. This concept extends the definition of personal data to entities other than individual persons.
- **Auditability**
  Auditability refers to the extent to which the quality of the data can be verified.
- **Credibility**
  Credibility is the degree to which data exhibit attributes that are considered true and believable by users in a particular context of use. Credibility encompasses the concept of authenticity (the truthfulness of origins, attributions, commitments).

Another problem based on the complexity of the task and environment is the potential loss of expressiveness of models. The loss of expressiveness of models is attributed to changes that are historically described by different terms and inconsistently used in the scientific literature. For reference, Moreno-Torres et al. [101] provide an overview of the various terms and their different definitions. In this paper, the two causes of loss of informativeness of models are described by the terms data drift and concept drift.

In data drift, a change in the independent variables (covariates/input characteristics) of the model leads to a change in the joint distribution of the input and output variables. AI components should be inspected for sources of data drift in the context of a safety risk analysis and adequate measures should be planned where necessary. Data drift is often tied to an incomplete representation of the input domain during training. Examples of this include, not accounting for seasonal changes in input data, unforeseen input by operators or the addition of new sensors that become available as input features. Naturally, data drift becomes an issue as soon as a model decays due to a change in the decision boundaries of the model.

Some examples of data drift can be attributed to missing the mark on the best practices in model engineering. Common examples include picking inappropriate training data, i.e., data whose distribution does not reflect the actual distribution encountered in the application context, or even omitting important examples in the training data. As such, these problem instances can be fixed by means of improved modelling and retraining.

Unfortunately, data drift is also caused by external factors, such as seasonal change or a change in process that induces data drift, e.g., replacement of a sensor with a new variant featuring a different bias voltage or encountering different lighting conditions in between training and previously unseen data. It can become necessary for the model to deal with data drift while already deployed, sometimes in cases where retraining is not feasible. In these cases, the model might be constructed in such a way that it is able to estimate correction factors based on features of the input data or allow for supervised correction. Overall, care must be taken to design the model to provide safe outputs, even if there are previously unknown inputs. It is important to understand that, even following proper model engineering practices, such as establishing a sufficiently diverse training dataset, there are no guarantees regarding the resulting model’s ability to generalise and adapt to the data encountered in production.

For reference, Amos and Storkey [102] provide illustrations for the most common sources of data drift and provide arguments for model improvements; even when the data drift can be categorised as a simple covariate shift and do not have any apparent effect on classification output, they can lead to simpler or computationally more efficient models. These performance considerations also translate into modern, deep neural networks [103].

Concept drift refers to a change in the relationship between input variables and model output and may be accompanied by a change in the distribution of the input data. Example: the output of a model might be used to gauge the acceptable minimal distance of an operator at runtime based on distance measurements obtained by a time-of-flight sensor (input data). If the accepted safety margins change due to external factors (e.g., increased machine speed not accounted for in the model), concept drift occurs while both processes and inputs have stayed the same.

Systems should incorporate forms of drift detection, distinguish drift from noise present in the system and should ideally adapt to changes over time. Potential approaches include models such as EDDM [104], detecting drift using support vector machines [105], or observing the inference error during training to allow for drift detection and potential adaptation while learning [106]. Furthermore, previous work quantifying drift in machine learning systems is available [107].

Drift is often handled by selecting subsets of the available training data or by assigning weights to individual training instances and then re-training the model. For reference, Gama et al. provide a comprehensive survey of methods that allow a system to deal with drift phenomena [108].

### 3.5. Degree of Transparency and Explainability

Often, aspects of traceability, explainability, reproducibility and general transparency are summarised under the term “transparency”. However, these terms must be clearly distinguished from one another. Transparency is the characteristic of a system that describes the degree to which appropriate information about the system is communicated to relevant stakeholders, whereas explainability describes the property of an AI system to express important factors influencing the results of the AI system in a way that is understandable for humans. For this reason, the transparency of a system is often considered a prerequisite for an explainable AI system. Even if this statement is not entirely correct, in relation to existing model-agnostic methods for increasing the explainability of neural networks, for example, a high degree of transparency nevertheless has a positive effect on the explainability of an AI system.

Information about the model underlying the decision-making process is relevant for transparency. Systems with a low degree of transparency can pose risks in terms of their fairness, security and accountability. Transparency is also a precondition on the reproducibility of the results of the system and bolsters its quality assessment.

The question of whether an AI system is recognisable as such for a user is also answered under this point. On the other hand, a high degree of transparency can lead to confusion due to information overload. It is important to find an appropriate level of transparency to provide developers with opportunities for error identification and correction, and to ensure that a user can trust the AI system.

During a risk assessment, it must be determined which information is relevant for different stakeholders and which risks can result from non-transparent systems for these stakeholders. In this case, a distinction can be made between two main groups of stakeholders:

- **The intended users:**
  Risks are examined that arise because the decisions and effects of the AI application cannot be adequately explained to users and other affected people.
- **Experts:**
  Risks are examined that arise because the behaviour of the AI application cannot be sufficiently understood and comprehended by experts such as developers, testers or certifiers.

Table 3 shows some relevant information for different stakeholders. For a developer, the information mentioned under the heading system is particularly relevant, whereas for auditors and certifiers, all the information mentioned is relevant. Users, of course, need to be educated about the nature of the system but only its basic functionality, so the information about the application is particularly interesting for this stakeholder group. However, information such as the objectives of the system and its known constraints are also of high importance for users, as these factors are crucial for safe operation.

With traditional software, the engineer’s intentions and knowledge are encoded into the system in a reversible process so that it is possible to trace how and why a particular decision was made by the software. This can be carried out, for example, by backtracking or de-bugging the software. On the other hand, decisions made by AI models, especially by models involving a high level of complexity, are more difficult to understand for humans, as the way knowledge is encoded in the structure of the model and the way decisions are made is rather different from the methods by which humans make decisions. This is especially true for models created with machine learning methods. The methods of deep learning (artificial neural networks) belonging to this category are of particular importance here, as they can sometimes become particularly complex, which means that they are usually almost impossible for a human to explain. Therefore, it is evident that, depending on the type of AI method used, a high degree of transparency does not always automatically lead to a high degree of explainability.

A high level of explainability protects against the unpredictable behaviour of the system but is often accompanied by a lower overall performance in terms of the quality of decisions. Here, a trade-off must often be made between explainability and the performance of a system.

In addition, the accuracy of the information about an AI system’s decision-making process is considered in each case. It is possible that a system can provide clear and coherent information about its decision-making process but that this information is inaccurate or incomplete.

Consequently, these aspects should also be included in the general evaluation of the AI system. That way, it is not only examined whether sufficient information about the system is available, but also if it is understandable for both experts and end users, thus delivering reproducible results for users. The question of whether an AI system is recognisable as such for a user is also answered under this point.

The degree of transparency and explainability can be divided into four categories, which are listed below in decreasing order of the degree of transparency and explainability:

1. Explainable:

The system provides clear and coherent explanations.

2. Articulable:

The system can extract the most relevant features and roughly represent their interrelationships and interactions.

3. Comprehensible:

The system is not capable of providing real-time explanations of system behaviour, but these are at least verifiable according to facts.

4. Black Box:

No information is available about how the system works.

Several evaluation concepts and strategies exist to judge the transparency or even explainability of an AI-based system, such as those reported in [109,110].

Additionally, empirical assessments of the decision process of complex models can be carried out, for example by inspecting a convolutional neural network through the visualisation of the components of its internal layers [111]. The goal is to make the network’s decision process more transparent by determining how input features affect the model output. Reviewing the output of a convolutional neural network by having its internal state inspected by a human expert is an approach that is extended in related work, such as [112,113,114]. Even when access to internal model states is completely unavailable, approaches such as RISE [115] can still provide insights into certain network types.

Even systems traditionally believed to be somewhat explainable with regard to inspection, e.g., decision trees, can quickly reach a complexity that defies understanding when deployed in real-world applications. In situations where an interpretable result is desired, tools, such as optimal classification trees [116] or born-again tree ensembles [117], can be applied to reduce complexity and allow for human expert review.

(See [118] for general thoughts on the relation between AI model types and their interpretability.)

Generally speaking, even if explainable AI is not immediately achievable and might not even be a prime concern when it comes to functional safety, a methodical and formally documented evaluation of model interpretability should be one of the assets employed in safety risk analysis, as this will aid comparability and model selection and can provide insights during a postmortem failure analysis.

### 3.6. Security

To assess the trustworthiness of an AI-based system, traditional IT security requirements also need to be considered. ISO/IEC 27001 [119], ISO/IEC 18045 [120] and ISO/IEC 62443 [121] already provide processes for the audit and certification of horizontal IT security requirements that are also applicable to AI-based systems.

In addition to following the best practices and observing existing standards for conventional systems, artificial intelligence comes with an intrinsic set of challenges that need to be considered when discussing trustworthiness, especially in the context of functional safety. AI models, especially those with higher complexities (such as neural networks), can exhibit specific weaknesses not found in other types of systems and must, therefore, be subjected to higher levels of scrutiny, especially when deployed in a safety-critical context.

One class of attacks on AI systems in particular has recently garnered interest: adversarial machine learning. Here, an attacker tries to manipulate an AI model to either cause it to malfunction, change the expected model output or obtain information about the model that would otherwise not be available to them.

When trying to manipulate a model, an attacker will typically either modify the input available to the model during inference or try to poison the learning process by injecting malicious data during the training phase. For example, it is possible to trick a model into outputting vastly different results by adding miniscule perturbations to the inputs. This noise is, in the case of input images, generally imperceptible to humans and may also be equally well hidden in numeric inputs. While these perturbations are typically non-random and carefully crafted by means of an optimisation process, it cannot be ruled out that hardware failures or system noise already present in the input can cause a non-negligible shift in model output; see [122], for example. Inputs modified in such a way are called adversarial examples. Adversarial examples translate somewhat well across different model architectures and intrinsic model components [123,124]. This, along with the fact that there are several well-known model architectures and pre-trained models available in so-called “model zoos”, makes the practical applicability of adversarial examples seem very likely.

Additionally, even a system that is seemingly resilient to the modification of its inputs, i.e., a system employing a local, non-cloud AI model directly connected to sensors, is not exempt from this attack vector. The feasibility of physical attacks on models, even if these are considered black boxes with no access to details, the availability of the internal model was already demonstrated by Kurakin et al. in 2017 [125]. More recently, Eykholt et al. [126] showed that it was possible to introduce adversarial examples into the forward inference process of a model by creating the aforementioned perturbations using physical stickers that are applied to objects and cause a vastly diverging classification result. In the examples presented, traffic signs were misclassified with a high success rate [126].

For systems with high demands for safety aspects, these weaknesses should be carefully addressed in terms of both random failures and systematic errors. Overall, failures should be addressed according to best practices, i.e., through hardening, robustification, testing and verification. Additionally, there are specific countermeasures available in the field of machine learning that can be applied to further mitigate the safety risks of AI-specific failure cases. Goodfellow et al. argue that a switch to models employing nonlinear components makes them less susceptible to adversarial examples; however, this comes at the cost of increased computational requirements [127]. Madry et al. [123] address the problem by examining and augmenting the optimisation methods used during training. Often, model ensembles are mentioned to create a more robust overall model through diversification. However, there are results that show that this might not sufficiently harden the system against adversarial examples (see He et al. [128]).

A first step in protecting against attacks on models might be to supply adversarial examples during training, in order to have the model encode knowledge about the expected output of those examples. This is called adversarial training.

The next natural avenue of action involves attempting to remove the artificially introduced perturbations. Some examples of this approach include the high-level representation guided denoiser (HGD) introduced by Liao et al. [129], MagNet, which aims to detect adversarial examples and revert them back to benign data using a reformer network [130] or Defense-GAN, which employs a generative adversarial network with similar goals [131]. It is worth mentioning that scenarios exist where both MagNet and Defense-GAN can fail (see [132]).

Furthermore, noting that the model types typically affected by adversarial attacks are generally robust against noise, several authors propose randomisation schemes to modify the input and increase robustness against malicious, targeted noise. Approaches include random resizing/padding [133], random self-ensembles (RSE) [134] and various input transformations such as JPEG compression or modifications of image bit depth [135]. While these methods can be surprisingly effective, recent results show that these transformations are not sufficient measures under all circumstances. In turn, if input transformations are used as a layer of defence against adversarial examples, the efficiency of said protective measures should be evaluated against examples generated using the Expectation over Transformation (EOT) algorithm presented in [136].

### 3.7. System Hardware

Of course, an AI model cannot make a course of decisions by itself; it depends on the algorithms, software implementing the AI model and hardware running the AI model. Faults in the hardware can violate the correct execution of any algorithm by violating its control flow. Hardware faults can also cause memory-based errors and interfere with data inputs, such as sensor signals, thereby causing erroneous results, or they can violate the results in a direct way through damaged outputs. This section describes some hardware aspects that can potentially affect the safety of an AI system. As a short summary, currently, we seem to need hardware that is as reliable as the hardware used for conventional systems. In general, hardware-related failures can be divided into three groups:

- Random hardware failures;
- Common cause failures;
- Systematic failures.

Similar to hardware used to execute conventional software, the hardware used to execute AI models also suffers from random hardware failure. These failures include short circuits or interruptions in conductor paths and component parts, short circuits between individual or multiple memory cells of variable and invariable memory, drifting clocks such as oscillators, crystals or PLLs (phase locked loops), and stuck-at errors or parasitic oscillations at the inputs or outputs of integrated circuits. Aside from these failure modes, soft errors can also have an effect. These types of random hardware failures describe unwanted temporary state changes in memory cells or logic components that are usually caused by high-energy radiation from sources such as alpha particles from package decay, neutrons and external EMI (electro-magnetic interference) noise, but can also be caused by internal crosstalk between conductor paths or component parts.

On the downside, when compared to conventional software, computations involving AI models require significantly larger amounts of data movements and arithmetic computations, depending on the types of models used. This may cause a higher probability of faults becoming actual failures, i.e., a higher probability of failure on demand per hour. Furthermore, the training of a model derived from a machine learning method and its execution usually takes place in different systems. Since both faults that occur during the training phase and in the operation of an AI system can affect the correct execution of the algorithm, both the system used for training and the system used for the execution of the AI algorithm are relevant.

In the context of artificial intelligence, GPUs (graphics processing unit), cloud computing or edge computing are the most common methods used for the execution and/or training of the AI algorithm.

Generally, GPUs share their error models with those of a central processing unit (CPU), which means that errors can occur in registers, RAM, address computation, programme counters and stack pointers, for example. The main difference between a CPU and a GPU is the memory and processor architecture. GPUs consist of many multiprocessors that each consist of several processor cores. Each of these multiprocessors is equipped with its own L1 cache, and these caches are not coherent. Compared to the L1 cache of a CPU, the GPU’s L1 cache is smaller but has a higher available bandwidth. Unlike the L1 cache, the L2 cache of a GPU is coherent but again smaller than the L2 or L3 cache of a CPU. Because of this, memory diagnostics measures are more challenging to implement on a GPU and an erroneous thread scheduler has a more critical effect.

The cloud computing method is characterised by the fact that it accesses a shared pool of configurable computing resources (such as servers, storage systems, applications and services) over the Internet, whereas edge computing is characterised by local computing power that is in close proximity to the attached devices and provides real-time capability. Since the exchange of data plays a central role in both technologies, it is of particular importance to perform an analysis of the possible faults of the network architectures used. A fault model for different networks includes, for example, errors such as data corruption, unintended repetition of messages, an incorrect sequence of messages, data loss, unacceptable delays, insertion of messages, masquerading and various addressing issues.

On the other hand, there are some reports (for example references [137,138,139]) suggesting that some internal redundancy of computations embedded in AI models will suppress the negative effects of soft errors to some extent. Despite this, it is difficult to predict the levels of such error suppression with any degree of reliability. The analysis of vulnerability factors for AI is an important aspect of random hardware failure in the context of functional safety.

Common cause failures can be created by AI at the hardware level, as the amount of power required to perform calculations and the loads on system design can vary depending on the data. As AI implementation typically requires more computation resources than the same functionality implemented in conventional software, careful hardware design and implementation is essential. With regard to common cause failures at the hardware level, there are no differences between conventional and AI-based hardware. A list of relevant common cause failures can be found in standards such as IEC 61508-2 [36] or ISO 26262-11 [140].

Systematic failures are also a cause of error when it comes to hardware systems for creating, maintaining, or running AI models. As the range of AI applications is expanding, embedded systems are also becoming increasingly important. In the training phase, the amount of data and computing power that is required to calculate the coefficients by a machine learning algorithm is very high and prevents the use of an embedded system during this phase. When the training phase is completed, the calculated coefficients are transferred to the target system. This asymmetry of machine learning methods means that much less computing power is required in the application phase; therefore, embedded systems can be suitable for this phase. However, there are some difficulties in implementing the training outcomes on a micro controller unit (MCU), micro processing unit (MPU) or digital signal processor (DSP), as many AI frameworks use Python as the description language, while the control programme of an embedded system is usually in C or C ++. Aside from this, incompatibilities with the read-only memory (ROM) and random access memory (RAM) management of an MCU, MPU or DSP are an additional cause of errors.

The use of parallel computing architectures also increases the risk of time-related programme errors, such as race conditions, deadlocks or heisenbugs.

A deadlock—also called a jam—is a state of processes in which at least two processes are waiting between each other for resources that are allocated to the other process. Thus, the execution of both processes is blocked.

A race condition is a constellation in which the result of an operation depends on the temporal behaviour of certain individual operations. They are a very difficult source of error to detect because the successful completion of the operation depends on chance.

A Heisenbug is a programme error (also called a bug) that is extremely difficult or impossible to reproduce. The defining characteristic of a Heisenbug is the extremely difficult recovery of the framework conditions necessary for the reproduction of the bug. The cause of this type of error is often the use of an analysis tool or debugger, as these can change the temporal framework conditions for the programme flow, and thus prevent the error from occurring. So, you either know the framework conditions without the bug or the bug without the framework conditions, hence the reference to Heisenberg’s uncertainty principle.

Some errors cannot be dedicated to a single pitfall, but instead arise from a combination of different ones. Other failures arise from common cause effects that are often not related to failures of single hardware components, but instead to other effects such as electromagnetic interference, temperature effects or decoding errors. Because of this, it is important to be aware of effects that might influence each other.

The following classification scheme of different integrity levels of the hardware is based on IEC 61508-2 [36]:

1. Quantified hardware, SIL 4 capable;
2. Quantified hardware, SIL 3 capable;
3. Quantified hardware, SIL 2 capable;
4. Quantified hardware, SIL 1 capable;
5. Non-quantified hardware proven in field of application;
6. Non-quantified hardware, proven in field of application;
7. Non-quantified hardware, recently released.

### 3.8. Technological Maturity

The technological maturity level describes how mature and error-free a certain technology is in a certain application context. If new technologies with a lower level of maturity are used in the development of the AI system, they may contain risks that are still unknown or difficult to assess. Mature technologies, on the other hand, usually have a greater variety of empirical data available, which means that risks can be identified and assessed more easily. However, with mature technologies, there is a risk that risk awareness decreases over time. Therefore, positive effects depend on continuous risk monitoring and adequate maintenance.

To determine the maturity level of a system, one can, for example, rely on the market’s experience with certain technologies or on a systematic analysis of the system’s behaviour in operation. Such an analysis is based on evidence of the system’s hours of operation in a similar application context, as well as the evaluation of the incidents reported with this system during this time.

The maturity of a technology for implementing an AI system can be classified as follows:

1. Current:
  The technology is currently supported and in use.
2. Preferred:
  The technology is already preferred for the implementation of most applications.
3. Limited:
  The technology is already operational for the implementation of a limited number of applications.
4. Strategic:
  The technology is likely to be operational only in the medium-to-long term.
5. Emerging:
  The technology is being researched and tested for possible future use.
6. Out of service:
  The technology is on the verge of no longer being used.

## 4. Conclusions

Artificial intelligence is still a very agile field of research that is making great progress. Essentially, we envisage three pillars derived from the rapid progress in the field of artificial intelligence within the last few years: The objective for this was the technical but also economic availability of a high computing power, which made it possible to significantly reduce the training times for deep neural networks [67,141,142]. The second pillar is the availability of large amounts of data, which makes the meaningful training of these deep neural networks possible [47,67,68,69], and the third pillar is the spread of the open-source idea [143,144,145]. This has not only made new methods, but also entire training algorithms or even complete models, quickly accessible to a broader public audience, which can thus be easily taken up by other working groups and directly used or further optimised.

All of these factors are still in place, which means that it can be expected that major advances will continue to be made in this field, resulting in the continued rapid market growth in artificial intelligence. Even though this technology has already established itself permanently on the market in some areas, the fields of application of artificial intelligence will be expanded in the future through the realisation of new innovative applications.

However, care must be taken to ensure that a human-centred approach is always adopted in the development of such systems. For this, compliance with basic safety principles is essential and must fulfil all the framework conditions for trustworthy AI.

This requires a precise understanding of the specific aspects of the individual artificial intelligence processes and their impact on the overall quality of the system in general, as well as its safety.

In particular, AI systems based on machine learning present new challenges for the security integrity of the system. Since their models are not developed directly based on the interpretation of a specification by human developers, but are indirectly derived from data, major difficulties exist, especially in creating the specification. Ashmore et al. (Ashmore, 2019) derived the risk source of an incomplete specification from this. Other works also name these and point out the problem of interpretability [146,147,148]. However, it can be stated here that the problem of incomplete specification is a consequence of the complexity of the task and operational environment of AI systems, which can thus be regarded as the actual original source of risk. Furthermore, the term interpretability is not defined in the basic standard for AI terminology [4], which instead defines the concept of explainability, also being reflected in the scientific discipline of XAI (explainable AI).

Many papers address the effectiveness of assurance cases for the quality assurance of AI systems [149,150,151]. An assurance case is defined as a reasoned, verifiable artefact that supports the assertion that a set of overlying assertions are satisfied, including a systematic argument with underlying evidence and explicit assumptions to support those assertions [152]. However, these works lack a detailed list of concrete criteria and only describe a few cases at a time, such as fairness [151], or only structure them on the basis of life-cycle phases according to standards such as the CRISP-DM [153], which means that comprehensive coverage of relevant risk areas cannot be achieved [21]. International standards for the AI field are still under development and usually only address partial aspects, such as the explainability [23] or controllability [24], of these systems, which are not applicable to the field of safety-related systems [25]. Legislative documents, on the other hand, only contain generic requirements that must first be interpreted and concretised [26]. There are only a few studies that deal with the definition and description of concrete sources of risk for AI, and they describe these only superficially and incompletely [12].

Therefore, a comprehensive and easily applicable list of new risks associated with AI systems, which also includes the field of safety-related systems, does not yet exist. Especially in the field of occupational safety and health, it is therefore necessary to identify these new risks and analyse the impact of AI features on risk management strategies, depending on the type of system under consideration and its context of application.

Therefore, this work attempts to provide a comprehensive collection of the relevant sources of risk for AI systems and to classify them in a meaningful taxonomy. The single sources of risk can be divided into risks that relate more to ethical aspects (fairness, privacy, degree of automation and control) and those that influence the reliability and robustness of the AI system (complexity of the intended task and usage environment, transparency and explainability, security, system hardware and technological maturity).

To facilitate the integration of these risk sources into a risk assessment of a system based on AI technologies, a risk management process for AI systems was further proposed and explained. With the help of this process, the individual sources of risk can be easily analysed and evaluated in terms of their criticality in order to define suitable risk mitigation measures at an early stage, which ultimately lead to a reliable and robust system and prevent unsafe failures of the AI system.

The individual sources of risk mentioned were evaluated through various steps. Not only were they compared with the partial results of other work, but requirements for trustworthy AI were also analysed so that it could be deduced from these whether the sources of risk mentioned were factors that influenced them. Finally, it was investigated whether the vulnerabilities derived from various accidents could have been revealed by these sources in advance through a risk assessment.

The description of the individual steps of the proposed risk assessment, as well as the description of the individual risk factors, provides the necessary basic understanding of these factors in order to achieve easy applicability, so that this work can provide guidance and assistance for the risk assessment of AI systems.

Even though an extensive evaluation of the sources of risk mentioned has been carried out, it must be noted that the taxonomy presented cannot claim ultimate and permanent completeness. This is due to the novelty of many AI methods and the high dynamics of research in the field of artificial intelligence. It cannot be ruled out that new incidents, such as accidents, will reveal new critical weaknesses or that new procedures will be developed that bring new aspects with them.

Furthermore, it can be discussed whether the allocation of the individual risk sources in the taxonomy is as valid as presented. It could be noted here that measures to ensure the fairness of a system also increase its robustness. However, we chose to assign fairness to the ethical aspects because at the risk assessment level this is a more ethical issue and only the measures to address possible discrediting outcomes ultimately intersect with the issue of task complexity and environment of use.

In order to ensure the completeness and validity of the taxonomy presented, it is absolutely necessary to continue monitoring research, especially in the field of the development of new methods in the field of AI, but also by constantly monitoring the development of new methods in the field of AI.

Furthermore, this work can only provide a basic understanding of the individual sources of risk. Therefore, it is necessary to use this taxonomy as a basis for further investigating each identified risk source in depth, to explore its causes and influences on the system and, in particular, to develop suitable measures for risk reduction.

It is also important to discuss the points mentioned above in the context of international standardisation, where there is a lack of requirements and especially of detailed guidance on the risk assessment of safety-related systems. This would be a great gain, especially in the area of testing and certification of such systems. The work presented can provide important input here. However, it can also provide important support for planners, developers, data scientists and other stakeholders.

## Figures and Tables

**Table 1:** Description of the seven degrees of automation [4]: no automation, assistance, partial automation, conditional automation, high automation, full automation and autonomy.

System	Level of Automation	Degree of Control	Comments
Autonomous	Autonomy	Human out of the loop	The system is capable of modifying its operation domain or its goals without external intervention, control or oversight
Heteronomous	Full automation	Human in the loop Human out of the loop	The system is capable of performing its entire mission without external intervention
	High automation	Human in the loop	The system performs parts of its mission without external intervention
	Conditional automation	Human in the loop	Sustained and specific performance by a system, with an external agent ready to take over when necessary
	Partial automation	Human in the loop	Some sub-functions of the system are fully automated while the system remains under the control of an external agent
	Assistance	Human in the loop	The system assists an operator
	No automation	Human in the loop	The operator fully controls the system

**Table 2:** Requirements for autonomous systems and the comparison to current AI systems.

	Autonomous System	AI System	
Consciousness			
Memory	Computer memory	No emotional memory	X
Learning	Machine learning	No intuitive learning	X
Anticipation	Predictive analysis	No intuition	X
Awareness	System status	No awareness of self	X
Ethics and morality	Functional morality	No full moral agency	X
Free will	Free decision making	Deterministic systems	X

**Table 3:** List of possible information to be communicated to different stakeholders.

System	Data	Application
Design decisions	Place of data collection	Type of application
Assumptions Models Algorithms Training methods Quality assurance processes Objectives of the system Known constraints	Time of data collection Reasons for data collection Scope of data collection Processing of data Data protection measures	Degree of automation Basis of results Basis of decisions User information

## Data Availability

Not applicable.
